# A robust adversarial learning framework for EEG emotion recognition

**DOI:** 10.3389/fneur.2026.1951986

**Published:** 2026-10-09

**Authors:** Hao Yang, Li Chen, Jianyong Gao, Junqiang Zhang, Jianfeng Zhang

**Affiliations:** Digitalization Division, State Grid Gansu Electric Power Company, Lanzhou, China

**Keywords:** adversarial training, distributionally robust optimization, domain generalization, electroencephalography, emotion recognition

## Abstract

**Introduction:**

Electroencephalography (EEG) provides an objective means of capturing neural patterns associated with emotion, but substantial inter-subject variability limits the ability of existing models to generalize to unseen subjects.

**Methods:**

We propose a robust adversarial domain generalization framework for cross-subject EEG emotion recognition. In the first stage, long short-term memory-based encoders are jointly trained to learn domain-invariant and domain-specific representations using reconstruction, emotion classification, and adversarial domain objectives. This process preserves emotion-relevant information while suppressing subject-specific distribution shifts. In the second stage, the invariant encoder and emotion classifier are further refined through distributionally robust optimization with gradient-guided feature augmentation. Theframework is evaluated on the AMIGOS and DREAMER datasets using leave-one-subject-out cross-validation.

**Results:**

The proposed method achieves mean accuracies of 69.94% and 68.97% and F1 scores of 0.59 and 0.65 on AMIGOS and DREAMER, respectively, outperforming the comparison methods on both datasets. Ablation results further show that adversarial learning provides the primary improvement in cross-subject generalization, while distributionally robust optimization offers complementary robustness.

**Discussion:**

These findings indicate that combining representation learning that is invariant across domains with distributionally robust optimization effectively reduces distribution shifts across subjects and improves EEG emotion recognition for unseen subjects.

## 1 Introduction

Emotions are psychological and physiological responses to internal and external stimuli, and they profoundly influence human cognition, perception, and everyday decision making (1). Effective emotion recognition is not only fundamental to interpersonal communication but also essential for enabling natural human-computer interaction (2). Altered emotional processing is a central feature of affective disorders such as major depressive disorder, and EEG can reveal the temporal neural dynamics underlying these abnormalities (3). Emotion recognition systems can interpret a user's affective state and enable artificial intelligence systems to adjust their feedback dynamically, offering broad application potential in healthcare, distance education, military operations, and other fields (4).

Among existing recognition modalities, facial expressions and speech provide intuitive cues but are easily affected by deliberate concealment or performance (5). Conversely, EEG provides a more reliable and objective basis for emotion recognition because it reflects cortical neuronal activity with high temporal resolution and is difficult to manipulate voluntarily (6). Compared with neuroimaging techniques such as functional magnetic resonance imaging, EEG equipment is easier to operate, less expensive, and better suited to wearable applications (7). In recent years, increasingly affordable, portable, and easy-to-use acquisition systems have brought a variety of wireless EEG devices to market (8).

Despite substantial progress in EEG emotion recognition, generalization in practical settings remains challenging. This limitation is primarily attributed to domain shift in EEG signals, which leads to substantial distributional discrepancies across subjects and recording conditions (9). Inherent differences in brain structure, such as cortical thickness; neural response mechanisms that vary by individual, including distinct emotional response patterns; and variation in acquisition procedures, such as electrode placement, jointly produce marked differences among subjects (10). Most existing methods nevertheless rely on a strict independent and identically distributed assumption and therefore struggle to handle heterogeneity across multiple sources commonly encountered in practice (11). A model trained on source domains may consequently fail to maintain its performance in an unknown target domain, severely limiting the practical deployment and widespread adoption of EEG emotion recognition technology (12).

Transfer learning has been widely adopted to alleviate distributional discrepancies across domains (13). Most domain adaptation methods aim to learn domain-invariant representations by aligning the distributions of source and target domains, yet they typically rely on access to target domain data during training (14, 15). This requirement is difficult to satisfy when target data are unavailable, restricted for privacy reasons, or must be processed online. Conversely, domain generalization learns stable and discriminative features from multiple source domains without using any target domain information, enabling the resulting model to remain reliable under unknown distributions (16, 17). This formulation better aligns with practical application requirements and offers an important path toward EEG-based emotion recognition that generalizes across scenarios. Nevertheless, effectively disentangling domain-related variations from complex EEG signals while preserving stable, emotion-relevant representations remains a critical challenge.

To address this problem, we develop a cross-subject EEG emotion recognition model in a domain generalization setting. The model combines adversarial training with distributionally robust optimization to remove domain-related interference from noisy EEG data and learn stable affective representations without access to target-domain data. The main contributions are as follows:

1. We propose an EEG emotion recognition model that integrates adversarial training with distributionally robust optimization. Adversarial learning extracts domain-invariant emotion features, while Wasserstein distributionally robust optimization further improves model generalization.
2. We design a two-stage optimization strategy. The first stage suppresses subject-specific shifts through adversarial domain training, and the second stage further refines the emotion classification branch through distributionally robust optimization, increasing robustness to cross-subject distribution shifts.
3. We conduct extensive experiments on the public AMIGOS and DREAMER datasets. Comparative experiments, ablation studies, and feature visualization analyses consistently verify the effectiveness and robustness of the proposed model for cross-subject emotion recognition.

The remainder of this paper is organized as follows. Section 2 reviews cross-subject EEG emotion recognition, domain generalization, and distributionally robust optimization. Section 3 describes the proposed model and its optimization procedure. Section 4 presents the experimental setup and evaluates the method on AMIGOS and DREAMER. Section 5 discusses the main findings, implications, and limitations. Section 6 concludes the paper.

## 2 Related work

### 2.1 Transfer learning in cross-subject EEG emotion recognition

Inter-subject variability in EEG signals creates a distribution shift between the training and test data, which can substantially reduce model generalization performance. To address this issue, researchers have applied transfer learning to cross-subject emotion recognition (18). Transfer learning methods for this problem fall into two main categories: domain adaptation and domain generalization.

Domain adaptation is a transfer learning strategy that addresses domain shift by mapping labeled source data and unlabeled target data into a shared feature space and minimizing their distributional differences (19). Domain adaptation has also been widely applied in affective computing (20). Ren et al. developed a multi-source domain adaptation framework that integrates pairwise transfer learning with selective source utilization to alleviate negative transfer in cross-subject EEG emotion recognition (21). Imtiaz et al. proposed a source-free unsupervised domain adaptation framework that combines adaptive regularization and local consistency learning to reduce domain shift and pseudo-label noise in cross-domain EEG emotion recognition (22). Although these domain adaptation methods have alleviated subject variability in EEG signals and achieved promising emotion recognition performance, they share an inherent limitation: they require access to target-domain data during training, which may introduce the risk of information leakage.

Domain generalization learns common, transferable representations from multiple source domains without accessing target-domain data, enabling robust prediction on unseen domains in practical scenarios (16). DICA learns a transformation kernel that maps multiple domains into a shared latent subspace with reduced distributional differences (23) and was first introduced by Ma et al. as a domain generalization baseline for cross-subject emotion recognition (24). Hagad et al. proposed a Context Generalized Model (CGM) to address limited EEG data and subject variability using deep neural networks (25). Disentangled learning seeks to decompose learned representations into functionally independent and interpretable components through an appropriate feature mapping (17). This paradigm has therefore been widely adopted to disentangle robust and generalizable representations. Wang et al. proposed denoising mixed mutual reconstruction (DMMR), which adopts a two-stage pretraining and fine-tuning strategy to learn subject-invariant EEG representations (26). Chen et al. proposed GDDN, which improves cross-subject EEG emotion recognition by disentangling common and subject-specific information from EEG connectivity and representations, while adaptively aggregating domain-specific predictions to enhance generalization to unseen subjects (27). Yang et al. proposed FDDGNet, which improves cross-subject EEG emotion recognition by disentangling emotion-relevant and subject-specific representations through orthogonal projection, information bottleneck learning, and signal reconstruction, thereby enhancing generalization to unseen subjects and datasets (28). Learning robust representations that generalize across domains remains challenging.

### 2.2 Distributionally robust optimization

Distributionally robust optimization (DRO) combines ideas from stochastic optimization and robust optimization. It uses distributional information from training data to avoid the excessive conservatism of conventional robust optimization while reducing errors caused by the strong assumptions about the true distribution required by stochastic optimization (29). A central issue in DRO is constructing an ambiguity set. Existing ambiguity sets are primarily based on moments or distances (30). Moment-based ambiguity sets include all probability distributions that satisfy prescribed moment constraints. Delage et al. investigated temporal uncertainty and formulated a distributionally robust optimization problem under first- and second-moment constraints, which they further reformulated into a finite-dimensional problem via its dual form (31). Distance-based ambiguity sets can take various forms. Hu et al. proposed a KL divergence-based DRO method that controls the ambiguity set size by bounding the distance between the empirical and true distributions (32). Sinha et al. proposed a Wasserstein distance-based distributionally robust optimization method and provided a practical training procedure for models using stochastic gradient descent and smooth activation functions (33). Distributionally robust optimization is commonly formulated as a minimax problem (34). An ambiguity set is first constructed by maximizing perturbations around the training data to cover the unknown true distribution. The model is then optimized by minimizing the worst-case risk over this set, thereby improving its generalization to test data. In this study, DRO is introduced to fine-tune the testing branch of the model, further enhancing its generalization ability.

## 3 Proposed framework

As shown in Figure 1, the proposed framework contains two training stages and five principal components: a domain-specific encoder, a domain-invariant encoder, a domain classifier, an emotion classifier, and a decoder. The first stage performs information-theoretic adversarial training to isolate domain-invariant emotion representations. The second stage applies distributionally robust optimization to the domain invariant emotion classification branch.

**Figure 1:**
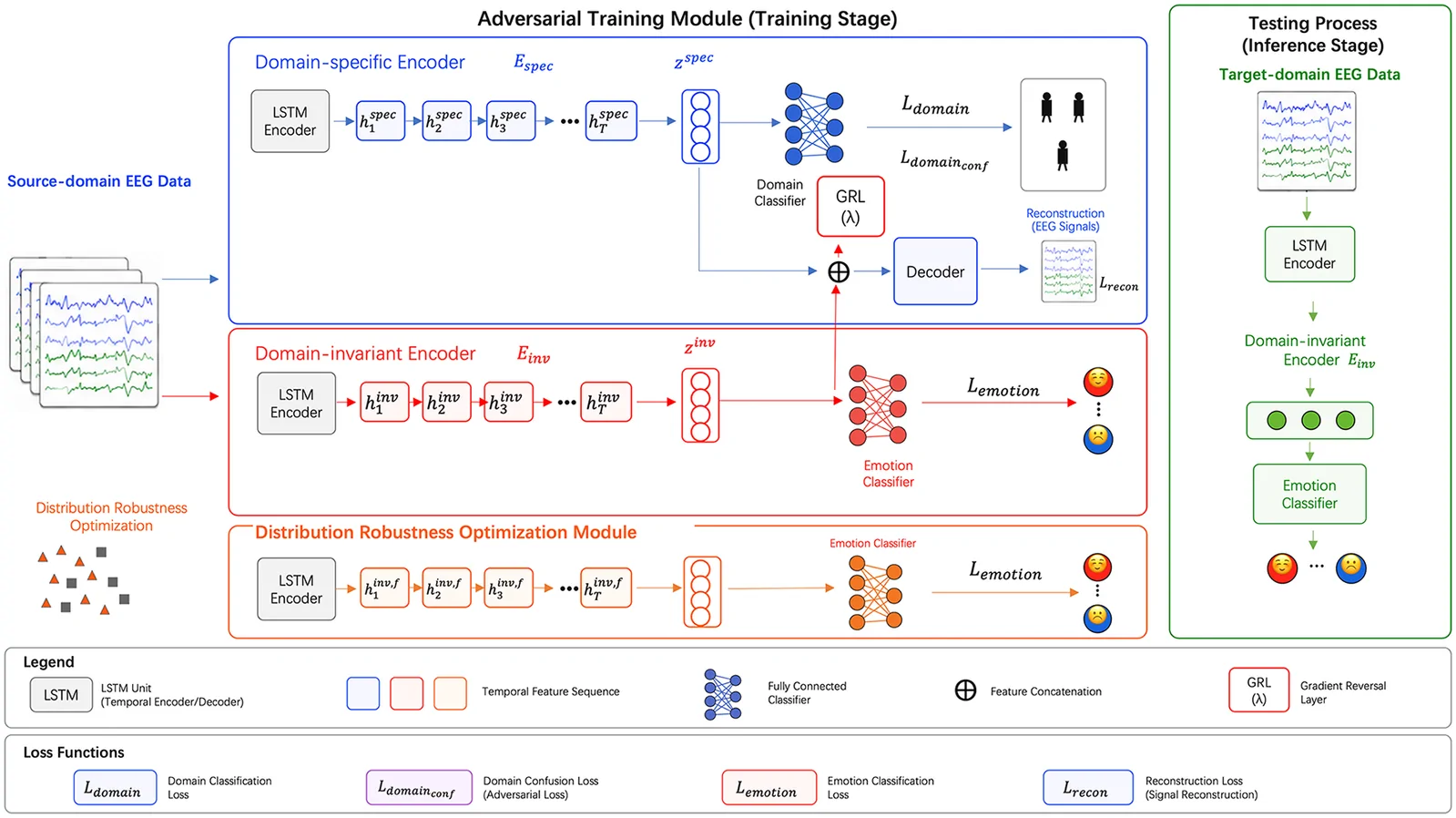
Overall architecture of the proposed EEG emotion recognition framework.

### 3.1 Problem formulation

To obtain stable cross-domain representations, latent EEG features are decomposed into domain-invariant and domain-specific components, and domain shift information is explicitly modeled to guide the learning of the invariant encoder. Let the source domain dataset be


$$\begin{array}{l} D_{s}={\left\{\left(x_{i}^{s},y_{i}^{s}\right)\right\}}_{i=1}^{N}, \end{array}$$ (1)

and let the target domain dataset be


$$\begin{array}{l} D_{t}={\left\{\left(x_{j}^{t},y_{j}^{t}\right)\right\}}_{j=1}^{M}. \end{array}$$ (2)

The source and target domain datasets are defined in Equations 1 and 2, respectively. The target domain labels $y_{j}^{t}$ are used only for evaluation and remain unavailable during model training. Throughout this manuscript, the superscripts *s* and *t* are reserved exclusively for samples from the source and target domains, whereas inv and spec denote domain invariant and domain specific representations, encoders, and parameters, respectively. The latent representation of an input *x*_*i*_ is denoted by $z_{i}=\left[z_{i}^{\text{inv}},z_{i}^{\text{spec}}\right]$, where $z_{i}^{\text{inv}}$ is the domain invariant component that is related to emotion but independent of domain, and $z_{i}^{\text{spec}}$ is the domain specific component that contains domain related information. We assume that *z*_inv_ and *z*_spec_ are stochastic functions of input *x* and domain label *d*, parameterized by θ_inv_ and θ_spec_, respectively. The class label *y* is modeled as a stochastic function of *z*_inv_ parameterized by θ_*c*_.

For a classification problem, let *l*(θ) denote a model loss and let L be the set of admissible losses. The parameter θ follows an unobservable true distribution *P*. For l∈L, the risk under *P* is as defined in Equation 3


$$\begin{array}{l} R\left(P,l\right)=E_{P}\left[l\left(\theta \right)\right], \end{array}$$ (3)

and the optimal risk is $R^{*}\left(P\right)=\underset{l\in L}{\inf}R\left(P,l\right)$. In practice, *P* is unknown and can only be inferred indirectly through the empirical distribution $\tilde{P}$ of the training samples. Replacing *P* directly with $\tilde{P}$ can propagate estimation bias into the final risk. We therefore use DRO to expand the neighborhood around the empirical distribution so that it is more likely to cover the true distribution and support less biased prediction.

### 3.2 Model architecture

EEG data have high temporal resolution and contain rich temporal information. LSTMs provide strong sequential feature extraction ability and use gated units to alleviate the long-range dependency problem of conventional recurrent neural networks. Their effectiveness in emotion recognition has also been demonstrated in earlier work. We therefore use LSTMs as the backbone of an encoder and decoder architecture comprising a domain-invariant encoder *E*_inv_, a domain-specific encoder *E*_spec_, and a decoder *F*.

Given an EEG sequence *x* = {*x*_1_, *x*_2_, …, *x*_*T*_}, each encoder uses the observation at time *t* and its previous hidden state to compute the current hidden state. Let $h_{t}^{\text{spec}}$ and $h_{t}^{\text{inv}}$ denote the hidden states produced at time step *t* by *E*_spec_ and *E*_inv_, respectively, where *t* = 1, 2, …, *T*. At the final encoding step, the hidden states produced by *E*_inv_ and *E*_spec_ are concatenated to initialize the decoder. Following the reverse reconstruction strategy used in sequence modeling, the decoder reconstructs the EEG sequence in reverse temporal order. A linear layer above the LSTM decoder maps its output to the dimensionality of the original EEG input, producing $\tilde{x}=\left\{{\tilde{x}}_{T},{\tilde{x}}_{T-1},\ldots ,{\tilde{x}}_{1}\right\}$.

The domain classifier *D* and emotion classifier *C* are stacks of fully connected layers. The emotion classifier receives domain-invariant features and predicts emotion labels. The domain classifier primarily receives domain shift features and predicts subject domains, thereby guiding *E*_spec_ to capture domain-specific information. Domain invariant features are also passed to the domain classifier through a gradient reversal layer. Reversed gradients guide *E*_inv_ to exclude domain shift components from its representation.

### 3.3 Stage I: adversarial training

The complete adversarial objective is given in Equation 4:


$$\begin{array}{l} J\left(\theta_{\text{inv}},\theta_{\text{spec}},\theta_{c};x,y,d\right)=\lambda_{r}I\left(x;z\right)+\lambda_{c}I\left(y;z_{\text{inv}}\right)\\ \;+\lambda_{d}\left(I\left(d;z_{\text{spec}}\right)-I\left(d;z_{\text{inv}}\right)\right), \end{array}$$ (4)

where *I*(·;·) denotes mutual information and λ_*r*_, λ_*c*_, and λ_*d*_ control the relative weights of the objective terms.

The term *I*(*x*; *z*) measures the mutual information between latent representation *z* and input *x*. Maximizing it encourages *z* to preserve information from the original data. We implement this objective with the encoder and decoder architecture and a reconstruction loss:


$$\begin{array}{l} L_{\text{recon}}=\frac{1}{N}\overset{N}{\underset{i=1}{\sum }}||x_{i}-F\left(E_{\text{inv}}\left(x_{i}\right),E_{\text{spec}}\left(x_{i}\right)\right){||}_{2}^{2}. \end{array}$$ (5)

This loss encourages the decoder to reconstruct the original EEG signal from both the invariant representation *z*_inv_ and shift representation *z*_spec_.

The term *I*(*y*; *z*_inv_) measures the mutual information between the domain invariant representation and emotion label *y*. Maximizing it encourages *z*_inv_ to retain class information. This relationship is enforced through the emotion classification cross-entropy loss:


$$\begin{array}{l} L_{\text{emotion}}=-\overset{N}{\underset{i=1}{\sum }}y_{i}\log C\left(E_{\text{inv}}\left(x_{i}\right)\right). \end{array}$$ (6)

The difference *I*(*d*; *z*_spec_)−*I*(*d*; *z*_inv_) encourages *z*_spec_ to preserve domain shift information while removing such information from *z*_inv_. This promotes shared representations that remain invariant across domains and reduces redundancy between *z*_inv_ and *z*_spec_. The domain classification loss is


$$\begin{array}{l} L_{\text{domain}}=-\overset{N}{\underset{i=1}{\sum }}d_{i}\log D\left(E_{\text{spec}}\left(x_{i}\right)\right). \end{array}$$ (7)

To minimize domain information in *z*_inv_, we use the gradient reversal strategy of an adversarial domain neural network. The corresponding classifier loss is


$$\begin{array}{l} L_{\text{domain\_conf}}=-\overset{N}{\underset{i=1}{\sum }}d_{i}\log D\left(E_{\text{inv}}\left(x_{i}\right)\right), \end{array}$$ (8)

whose gradient is reversed before it reaches *E*_inv_. The complete Stage I loss is


$$\begin{array}{l} L_{\text{adv}}=\lambda_{r}L_{\text{recon}}+\lambda_{c}L_{\text{emotion}}+\lambda_{d}\left(L_{\text{domain}}+L_{\text{domain\_conf}}\right). \end{array}$$ (9)

### 3.4 Stage II: distributionally robust optimization

To quantify the sensitivity of risk to the unknown true distribution *P*, we need a distance between probability distributions. We use the Wasserstein distance. For any *p*∈[1, ∞), the Wasserstein distance between two probability distributions *Q* and *Q*′ on ℝ^*m*^ is defined in Equation 10:


$$\begin{array}{l} W_{p}\left(Q,Q^{\prime }\right)={\left(\underset{\pi \in \Pi \left(Q,Q^{\prime }\right)}{\inf}\int_{{\mathbb{R}}^{m}\times {\mathbb{R}}^{m}}||\xi -\xi^{\prime }{||}^{p}\pi \left(d\xi ,d\xi^{\prime }\right)\right)}^{1/p}, \end{array}$$ (10)

where ||·|| is a norm on ℝ^*m*^ and Π(*Q, Q*′) is the set of joint distributions over ξ and ξ′ with marginals *Q* and *Q*′, respectively. Unlike KL and Jensen–Shannon divergences, the Wasserstein distance is continuous and can quantify differences between distributions with disjoint support, providing useful gradient information.

We construct a Wasserstein ball of radius ε centered on the empirical training distribution. Because the training data are samples from the true distribution, we assume the discrepancy between the empirical and true distributions is finite. An appropriate ε can therefore produce a Wasserstein ball that contains the true distribution. Optimizing the model for the worst-case distribution within this ball then improves its performance under the unknown true distribution. The choice of ε directly affects model performance. Using a Lagrangian formulation, the robust optimization problem and its inner maximization term are given in Equations 11 and 12, respectively:


$$\begin{array}{l} \underset{\theta \in \Theta }{\min}F\left(\theta \right):=\underset{P}{\sup}\left\{E_{P}\left[\ell \left(\theta ;Z\right)\right]-\gamma W_{c}\left(P,P_{0}\right)\right\}=E_{P_{0}}\left[\phi_{\gamma }\left(\theta ;Z\right)\right], \end{array}$$ (11)

where


$$\begin{array}{l} \phi_{\gamma }\left(\theta ;z_{0}\right):=\underset{z\in Z}{\sup}\left\{\ell \left(\theta ;z\right)-\gamma c\left(z,z_{0}\right)\right\}. \end{array}$$ (12)

This leads to a stochastic gradient procedure with alternating maximization and minimization. The maximization step perturbs each original sample using the loss gradient to construct an ambiguity set, and the minimization step optimizes the model loss on that set.

Unlike traditional data augmentation methods such as Mixup, which create synthetic samples by combining existing observations and their labels, DRO explicitly considers uncertainty in the data distribution. Mixup mainly increases data diversity within the space defined by combinations of observed samples, whereas our DRO strategy constructs a Wasserstein ambiguity set around the empirical source domain distribution and optimizes the model against unfavorable distributional variations within this set. The inner maximization identifies perturbations that increase the prediction loss, while the outer minimization encourages the model to remain robust to these variations. This mechanism is particularly relevant to subject-independent EEG emotion recognition, where unseen subjects may exhibit distribution shifts that are difficult to capture through simple combinations of training samples. Therefore, DRO complements the representation learning in Stage I by improving robustness to potential distribution shifts and enhancing generalization to unseen distributions.

Stage II DRO is applied only to the branch consisting of the domain-invariant encoder and emotion classifier. Adversarial samples are generated by backpropagation:


$$\begin{array}{l} x_{i}^{f}=x_{i}+\varphi \mathrm{sign}\left(\nabla_{x}L\left(C\left(E_{\text{inv}}\left(x_{i}\right)\right),y_{i}\right)\right), \end{array}$$ (13)

where φ controls perturbation strength. After the maximization step, the model is optimized on the ambiguity set by minimizing


$$\begin{array}{l} L_{\text{dro\_emotion}}=-\overset{N}{\underset{i=1}{\sum }}y_{i}\log C\left(E_{\text{inv}}\left(x_{i}^{f}\right)\right). \end{array}$$ (14)

Improving predictive accuracy on these perturbed samples increases the model's robustness and generalization to unseen or out-of-distribution data.

### 3.5 Optimization procedure

Training is performed in two stages. In Stage I, the inputs are standardized and passed through *E*_inv_ and *E*_spec_. Their representations are used for reconstruction, domain classification, domain confusion, and emotion classification. The domain invariant encoder *E*_inv_, domain specific encoder *E*_spec_, decoder *F*, domain classifier *D*, and emotion classifier *C* are jointly optimized using $L_{\text{adv}}$ in Equation 9; thus, no component is frozen during Stage I. In Stage II, adversarial training remains active. Each epoch first performs a standard Stage I update with $L_{\text{adv}}$, during which all five modules continue to be updated. We perform an additional DRO update every 20 epochs. The network parameters are held fixed while gradient ascent according to Equation 13 generates the perturbed samples $x_{i}^{f}$. During the subsequent DRO minimization step, *E*_spec_, *F*, and *D* are temporarily frozen, whereas only *E*_inv_ and *C* are updated using $L_{\text{dro\_emotion}}$ in Equation 14. The frozen modules are reactivated for the next adversarial training update. Therefore, Stage II enhances rather than replaces the adversarial representation learning established in Stage I. During inference, only *E*_inv_ and *C* are retained to predict the emotion label of each target domain sample; *E*_spec_, *F*, and *D* are not used. Algorithm 1 summarizes the complete training and inference procedure.

Algorithm 1 **Adversarial training and distributionally robust optimization.**

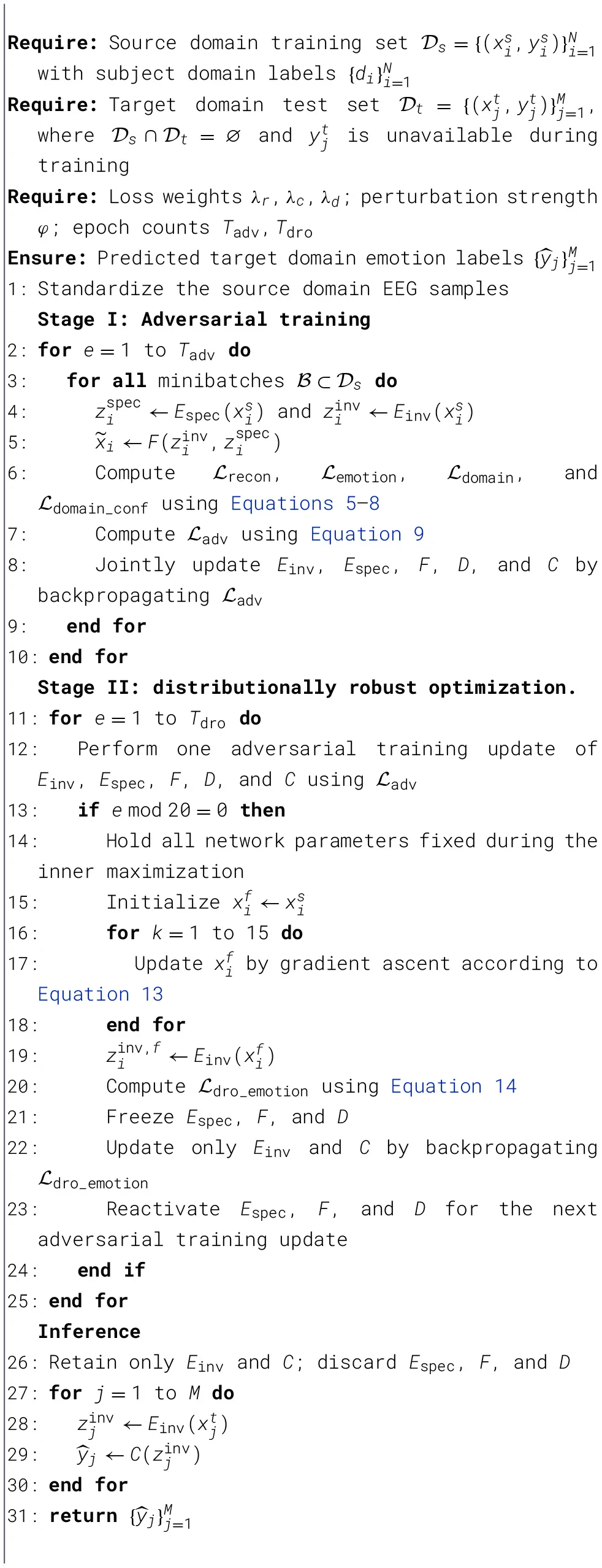

## 4 Experiments

### 4.1 Datasets and experimental settings

#### 4.1.1 Datasets

We use the public multimodal affective datasets AMIGOS (35) and DREAMER (36) to evaluate the proposed model for cross-subject EEG emotion recognition.

AMIGOS contains physiological signals (EEG, electrocardiography, and electrodermal activity) and video recordings from 40 participants in individual and social settings. It also provides self-reported and externally assessed emotion labels and personality measures. We use only EEG data from the individual setting. The EEG signals in the AMIGOS dataset were recorded using a 14-channel Emotiv EPOC headset at a sampling rate of 128 Hz. The stimuli comprise 16 short videos lasting 51–150 s and four excerpts from longer films, selected to cover all four quadrants of the valence–arousal space. Self-assessment labels include valence, arousal, and dominance. Six participants are excluded because of anomalous valence labels or corrupted data, leaving short video EEG recordings from 34 participants.

DREAMER contains EEG signals from 23 participants recorded with an Emotiv EPOC headset. Eighteen film clips were used to elicit nine basic emotions, and neutral clips were shown before and after each stimulus to provide physiological baselines. After viewing each clip, participants rated valence, arousal, and dominance using the Self-Assessment Manikin. We used the final 60 s of EEG recorded during each stimulus and retained data from all participants.

#### 4.1.2 Preprocessing

For both datasets, EEG preprocessing and label construction were performed under a unified processing protocol. Raw EEG recordings were first converted to MNE format and band-pass filtered from 4 to 40 Hz to suppress low-frequency drift and high-frequency noise. Bad channels were identified by visual inspection and repaired using spatial interpolation, while segments containing excessive bad channels were discarded. Independent component analysis was then applied to remove ocular movement and blink artifacts, followed by average referencing to reduce reference-electrode bias. The preprocessed continuous recordings were then divided into nonoverlapping 1-s windows as model inputs. Each EEG segment was assigned a binary valence label according to the corresponding affective rating. For AMIGOS, valence scores below 5 were labeled as low valence, whereas scores equal to or above 5 were labeled as high valence. For DREAMER, we adopted a threshold of 3, with scores below 3 classified as low valence and scores equal to or above 3 as high valence.

#### 4.1.3 Evaluation protocol and implementation

We evaluate generalization to unknown domains using leave-one-subject-out cross-validation on both datasets. In each fold, all samples from one participant form the test set and samples from all remaining participants form the training set. The test subject never participates in training, and the procedure is repeated for every subject. We use accuracy andF1 score as evaluation metrics.

The loss weights are λ_*r*_ = 1, λ_*c*_ = 1, and λ_*d*_ = 2. The batch size is 512, the adversarial training stage runs for 3,000 epochs, and the DRO stage runs for 2,000 epochs. Optimization uses Adam with a learning rate of 1 × 10^−4^. Each LSTM has a hidden state dimension of 512. The perturbation strength φ increases linearly from 0 to 0.02 over training. All experiments are implemented in PyTorch 2.1 (Meta AI, Menlo Park, CA, United States) and run on a server equipped with an NVIDIA GeForce RTX 4090 GPU.

### 4.2 Comparative experiments

We compare the proposed method with nine representative methods:

1. **Support vector machine (SVM):** a conventional baseline using manually designed differential entropy features.
2. **EEGNet** **(****37****):** a compact convolutional neural network designed specifically for EEG and used here as the deep learning baseline.
3. **DG-DANN** **(****38****):** a domain generalization variant of the domain adversarial neural network.
4. **DResNet** **(****24****):** a domain residual network that combines ideas from DG-DANN and domain separation networks. It adds an encoder that explicitly extracts domain shift information and uses adversarial training to help the model discover shared features.
5. **KA-CRNN** **(****39****):** a knowledge guided attention recurrent convolutional neural network. Personal information such as personality, sex, and age guides attention weight learning and is combined with EEG to improve cross-subject prediction.
6. **SICR-BCI** **(****40****):** a mutual information-driven framework for learning domain-invariant and class-relevant deep representations. It separates intermediate features into task-relevant and task-irrelevant components and enriches general representations across subjects through mutual information-based learning.
7. **BioDG** **(****41****):** A ResNet 18 backbone is augmented with one-dimensional feature extraction branches attached to three intermediate layers, and the resulting branch features are concatenated with the final backbone representation.
8. **EEG-DG** **(****42****):**A multi-source domain generalization framework is developed based on joint distribution stability. By enforcing invariance in both marginal and conditional distributions across source domains, the framework learns feature representations that remain stable across domains.
9. **NSAL-DGAT** **(****43****):** This domain adaptation method exploits the semantic topological relationships across domains to capture target semantic information, thereby improving classifier adaptation to target instances.

Table 1 summarizes the mean LOSO performance of the ten methods. On AMIGOS, the proposed method achieves the best results, with an accuracy of 69.94% and an F1 score of 0.59. Its accuracy exceeds those of the conventional SVM and EEGNet baselines by 19.36 and 17.88 percentage points, respectively, while its F1 score is higher by 0.17 and 0.18. Among the comparison methods, SICR-BCI gives the second-best accuracy of 67.08%, whereas EEG-DG gives the second-best F1 score of 0.58. The proposed method improves on these two strongest competing results by 2.86 percentage points in accuracy and 0.01 in F1. Compared with the newly included BioDG and EEG-DG methods, it provides accuracy gains of 15.14 and 5.58 percentage points, respectively. It also outperforms NSAL-DGAT by 8.31 percentage points in accuracy and 0.08 in F1.

**Table 1:** Comparative results on the AMIGOS and DREAMER datasets (mean).

Dataset	Model	Mean accuracy (%)	F1 score
AMIGOS	SVM	50.58	0.42
	EEGNet	52.06	0.41
	DG-DANN	60.02	0.49
	DResNet	64.01	0.53
	KA-CRNN	66.21	0.55
	SICR-BCI	67.08	0.57
	BioDG	54.80	0.53
	EEG-DG	64.36	0.58
	NSAL-DGAT	61.63	0.51
	**The proposed method**	**69.94**	**0.59**
DREAMER	SVM	55.45	0.48
	EEGNet	54.63	0.52
	DG-DANN	61.60	0.55
	DResNet	63.61	0.56
	KA-CRNN	64.21	0.61
	SICR-BCI	66.26	0.61
	BioDG	56.89	0.50
	EEG-DG	62.13	0.48
	NSAL-DGAT	62.89	0.46
	**The proposed method**	**68.97**	**0.65**

Bold values indicate the best performance for each dataset and evaluation metric.

The proposed method maintains its advantage on DREAMER, reaching an accuracy of 68.97% and an F1 score of 0.65. SICR-BCI is the strongest competing method in accuracy at 66.26%, giving the proposed method a gain of 2.71 percentage points. KA-CRNN and SICR-BCI share the second-best F1 score of 0.61, which is 0.04 below the proposed method. Relative to BioDG, EEG-DG, and NSAL-DGAT, the proposed method improves accuracy by 12.08, 6.84, and 6.08 percentage points and improves F1 by 0.15, 0.17, and 0.19, respectively. The consistent gains on both datasets indicate that the proposed framework improves class-balanced recognition as well as overall prediction accuracy.

Figure 2 complements the mean results by showing the distributions across test subjects, with outlier markers omitted for visual clarity. The proposed method has the highest median accuracy and F1 score in all four panels. On AMIGOS, its accuracy box is located above those of all comparison methods and is relatively compact. EEG-DG provides the closest F1 distribution among the newly added methods, whereas BioDG has a substantially lower accuracy median despite a moderate F1 median. On DREAMER, EEG-DG and NSAL-DGAT show competitive accuracy medians but noticeably lower F1 medians, indicating less balanced recognition across emotion classes. BioDG performs worse in both central accuracy and F1. Conversely, the proposed method maintains high medians and upper quartiles for both metrics on the two datasets. This agreement between the distributions across subjects in Figure 2 and the mean values in Table 1 shows that the overall gains are not caused by only a small number of favorable test folds.

**Figure 2:**
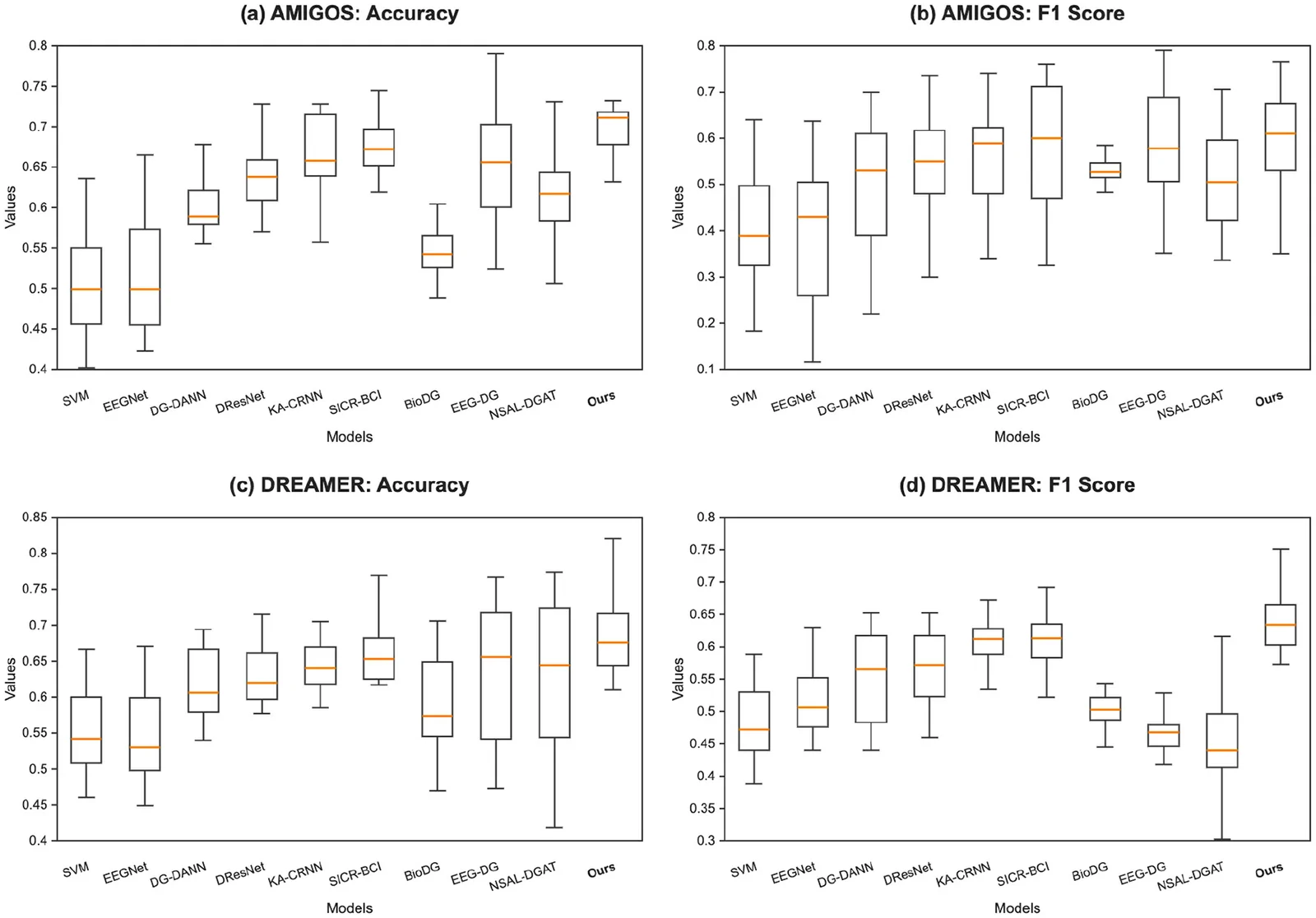
Cross-subject distributions of accuracy and F1 score for the comparison methods: **(a)** accuracy on AMIGOS, **(b)** F1 score on AMIGOS, **(c)** accuracy on DREAMER, and **(d)** F1 score on DREAMER. The proposed method is denoted by “ours.”

Many adversarial transfer learning methods use a similarity loss to separate domain-invariant and domain-shift representations, but this term reduced performance in our experiments. For complex EEG signals, directly enforcing feature separation may suppress emotion-relevant information, particularly because the classifier further transforms encoder features through two fully connected layers. Using the adversarial loss alone therefore produced more effective invariant representations and reduced the risk of negative transfer.

### 4.3 Ablation study

To isolate the contributions of the two training stages, we evaluate three configurations in addition to the complete proposed method: a baseline containing only the LSTM encoder and decoder and emotion classifier, a configuration using adversarial training without DRO, and a configuration using DRO without adversarial training. The results are summarized in Table 2.

**Table 2:** Ablation results on the AMIGOS and DREAMER datasets (mean).

Configuration	AMIGOS		DREAMER	
	Accuracy (%)	F1 score	Accuracy (%)	F1 score
Baseline	52.15	0.39	49.78	0.48
Adversarial training only	65.65	0.57	66.18	0.60
DRO only	56.37	0.43	55.22	0.54
**The proposed method**	**69.94**	**0.59**	**68.97**	**0.65**

Bold values indicate the best performance for each dataset and evaluation metric.

As shown in Table 2, adversarial training alone improves accuracy over the baseline by 13.50 percentage points on AMIGOS and 16.40 points on DREAMER, while increasing F1 score by 0.18 and 0.12, respectively. These substantial gains confirm that suppressing subject-specific shifts is the primary contributor to cross-subject generalization. Applying DRO alone yields smaller but consistent improvements, increasing accuracy by 4.22 points on AMIGOS and 5.44 points on DREAMER and increasing F1 score by 0.04 and 0.06. When both stages are combined, the proposed method further improves accuracy over adversarial training alone by 4.29 points on AMIGOS and 2.79 points on DREAMER, with corresponding F1 gains of 0.02 and 0.05. These results show that DRO provides complementary robustness after domain-invariant emotion features have been learned through adversarial training.

### 4.4 Feature visualization analysis

We use t-distributed stochastic neighbor embedding (t-SNE) (44) to examine the deepest representations learned by the proposed method near the classification output. For each dataset, we obtain the visualization from one representative leave-one-subject-out training-test split. The DREAMER split is selected to reduce the influence of severe class imbalance because its two emotion classes are relatively balanced. Figure 3 shows the resulting two-dimensional embeddings, where blue and red points denote low and high valence samples, respectively.

**Figure 3:**
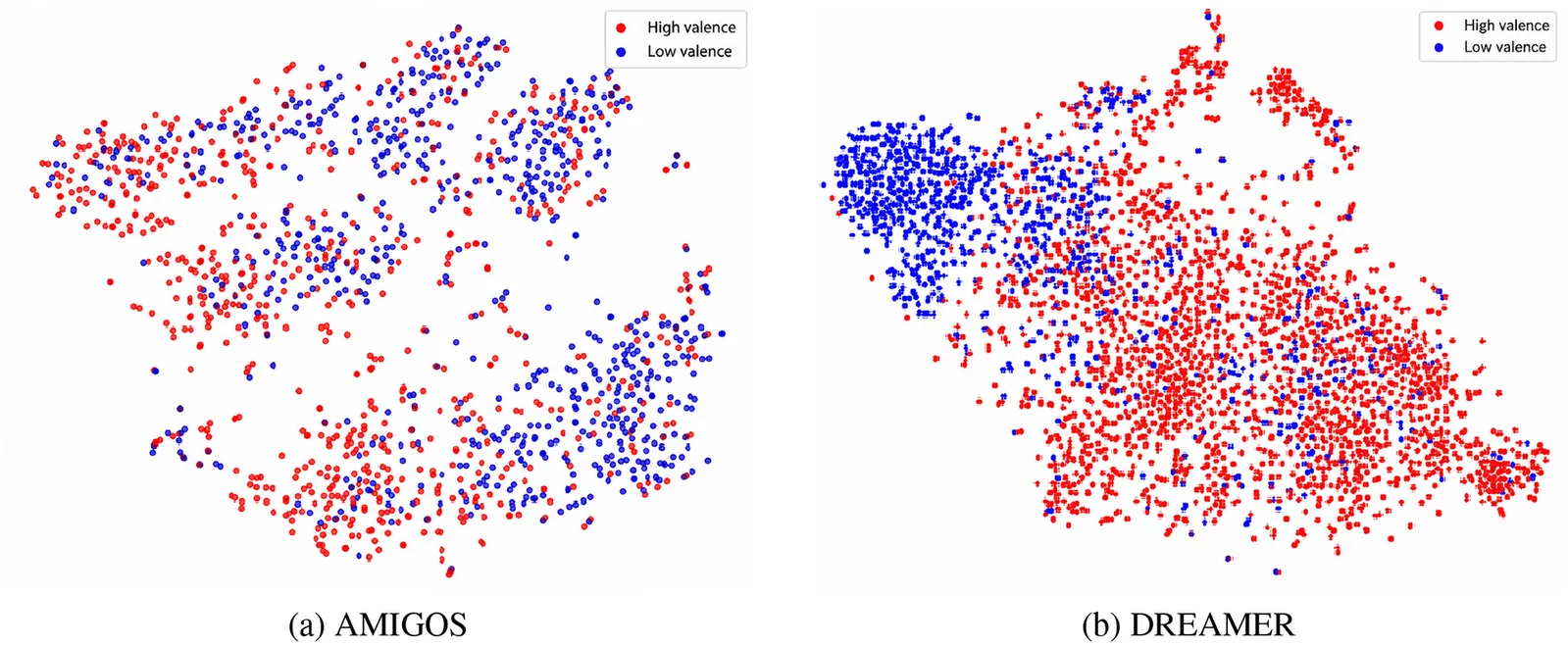
t-SNE visualization of the deepest feature representations learned by the proposed method for representative LOSO training test splits from **(a)** AMIGOS and **(b)** DREAMER. Blue and red points represent low- and high-valence samples, respectively.

As shown in Figure 3a, the AMIGOS representation forms several local clusters dominated by one class. Although a limited number of samples from the two classes remain interspersed, most regions are dominated by one class, indicating that the learned representation retains discriminative emotion information while reducing variation among subjects. In Figure 3b, the DREAMER features exhibit a different global geometry: low valence samples are concentrated mainly in the upper left region, whereas high valence samples occupy most of the central, lower, and right regions. Some mixing remains near the transition between these regions, reflecting the intrinsic overlap and variability of EEG emotion patterns. Nevertheless, the consistent emergence of structure associated with each class in both datasets suggests that adversarial suppression of domain-shift information, followed by DRO-based refinement, enables the proposed method to learn transferable and discriminative representations across unseen subjects.

## 5 Discussion

### 5.1 Main findings

This study developed a two-stage training framework that combines adversarial learning of domain-invariant representations with distributionally robust optimization for cross-subject EEG emotion recognition. Under the same LOSO evaluation protocol, the proposed method achieved the best overall performance among the compared methods, with an accuracy of 69.94% and an F1 score of 0.59 on AMIGOS, and an accuracy of 68.97% and an F1 score of 0.65 on DREAMER. The distributions across subjects in Figure 2 further show that these improvements are reflected in consistently high median performance rather than being driven by only a few favorable test folds. These findings indicate that the proposed framework improves overall recognition performance and enhances generalization to unseen subjects.

### 5.2 Contribution of adversarial representation learning

The ablation results demonstrate that adversarial training is the primary contributor to cross-subject generalization. Compared with the baseline, adversarial training alone improves accuracy by 13.50 percentage points on AMIGOSand 16.40 points on DREAMER, with corresponding F1 improvements of 0.18 and 0.12. This result supports the motivation for explicitly separating domain-invariant and domain-specific information. The domain-specific encoder preserves variation related to subjects, whereas the gradient reversal mechanism discourages the domain-invariant encoder from retaining information that enables subject discrimination. Consequently, the emotion classifier receives representations that preserve affective information while being less sensitive to distribution shifts among subjects.

The feature visualizations provide additional qualitative support for this interpretation. Although some overlap remains because of the intrinsic variability of EEG emotion responses, the learned representations form regions associated with each class in both datasets. This suggests that adversarial suppression of subject-related information does not remove all discriminative emotional structure. Instead, it produces representations that remain informative for emotion classification across unseen subjects.

### 5.3 Complementary role of distributionally robust optimization

DRO provides a complementary improvement after the domain-invariant representation has been established. Compared with adversarial training alone, combining the two stages improves accuracy by a further 4.29 percentage points on AMIGOS and 2.79 points on DREAMER, while increasing the F1 score by 0.02 and 0.05, respectively. By contrast, DRO alone produces smaller improvements over the baseline. These results indicate that robust optimization is more effective when applied to a representation that has already suppressed substantial subject-specific variation.

Unlike augmentation strategies based on fixed transformations or linear interpolation, the DRO stage approximately optimizes the model against loss-increasing samples within an ambiguity set around the empirical distribution. The resulting perturbations emphasize unfavorable directions for the current classifier and refine the domain-invariant encoder and emotion classifier accordingly. Therefore, Stage II does not replace the adversarial learning performed in Stage I; it strengthens the learned emotion branch against residual and previously unobserved distribution shifts.

### 5.4 Relation to recent research trends and practical implications

Recent cross-subject EEG emotion recognition studies have increasingly focused on domain adaptation, domain generalization, invariant representation learning, and robust optimization. The proposed method follows this direction but differs from methods that require access to target domain data during training. In the LOSO setting used here, the test subject remains entirely unavailable during model optimization. The observed improvements therefore reflect generalization to an unseen subject rather than adaptation to a known target distribution.

Nevertheless, performance values reported by different studies should be compared cautiously because they may depend on dataset partitions, preprocessing pipelines, label thresholds, segment lengths, and whether target domain information is available during training. For this reason, the most reliable evidence comes from controlled comparisons in which all methods use the same preprocessing and LOSO protocol. From an application perspective, reducing dependence on calibration for each subject may support more practical affective computing systems, although further validation is required before the framework can be considered for practical or clinical use.

### 5.5 Limitations

Several limitations should be acknowledged. First, the evaluation is restricted to the AMIGOS and DREAMER datasets, both of which contain EEG recordings with a limited number of channels. Additional datasets acquired with different devices, electrode configurations, and experimental protocols are needed to evaluate generalization across acquisition conditions. Second, the present study considers only binary valence classification. The framework's effectiveness for arousal, dominance, and multiclass emotions remains to be investigated. Third, the experiments evaluate cross-subject generalization within each dataset but do not include evaluation across datasets. Such evaluation would introduce larger differences in recording devices, preprocessing protocols, stimulus materials, and participant populations. Future work should investigate adaptive uncertainty sets, computationally efficient robust optimization, additional affective dimensions, and validation on independent practical EEG cohorts.

## 6 Conclusion

This study presents an EEG emotion recognition framework in which adversarial training learns transferable domain-invariant representations and periodic DRO updates further improve robustness to unfavorable distribution shifts. Results on AMIGOS and DREAMER demonstrate that the two stages provide complementary benefits for cross-subject recognition. These findings support adversarial representation learning and robust optimization as a promising strategy for generalization to unseen subjects.

## Data Availability

The AMIGOS and DREAMER datasets analyzed in this study are available from the sources described in their respective dataset publications (35, 36), subject to the applicable access conditions. No new data were generated in this study. Further inquiries can be directed to the corresponding author.
